# Whole-genome sequence of *Bacillus subtilis* ka15: a potent starter isolated from *Kawal*, a fermented condiment

**DOI:** 10.1128/mra.00055-25

**Published:** 2025-07-22

**Authors:** Blaise Waongo, François Tapsoba, Pingdwindé Marie Judith Samadoulougou-Kafando, Libère Ndayishimiye, Hama Cissé, Abakar Idriss Lawane, Donatien Kaboré, Aly Savadogo

**Affiliations:** 1 Laboratory of Applied Biochemistry and Immunology, University Joseph KI-ZERBO 107750, Ouagadougou, Burkina Faso; 2 Département Technologie Alimentaire (DTA), Centre National de la Recherche Scientifique et Technologique (CNRST), Institut de Recherche en Sciences Appliquées et Technologies (IRSAT) 206843, Ouagadougou, Burkina Faso; 3 National Key Laboratory of Agricultural Microbiology, Key Laboratory of Environment Correlative Dietology, College of Food Science and Technology, Huazhong Agricultural University 540859, Wuhan, Hubei, China; 4 Laboratoire de Recherche, de diagnostic et d’Expertise Scientifique, Université de N’Djamena, N’Djamena, Tchad; University of Guelph, Guelph, Canada

**Keywords:** genomics, *Bacillus subtilis*, *Kawal*, starter, fermented condiment

## Abstract

*Bacillus subtilis* named ka15 was isolated from *Kawal*, a fermented condiment produced from the *Senna obtusifolia* leaves. The genomic sequence size is 4,312,934 bp with 244 contigs, 43.5% average content of GC, 82 tRNAs, 28 rRNAs, 5 ncRNAs and 4,588 coding genes.

## ANNOUNCEMENT

*Bacillus subtilis* is recommended as a starter in the production of African condiments and fermented foods (1, 2). It has antimicrobial, anticancer, antioxidant, and vitamin production properties (3). However, some *Bacillus* species probiotics or starter cultures that are sold have antibiotic resistance genes (4). This is why the experimental and *in silico* analyses of these strains are necessary in order to assess their bioactivity and toxicity before their use in food. The present work provides a genome sequence project of *Bacillus subtilis* ka15, which was isolated from *Kawal*.

The *Kawal* is a fermented condiment extracted from the natural alkaline fermentation of the *Senna obtusifolia* leaves (5). The *Bacillus subtilis* ka15 isolate has been isolated from a *Kawal* sample and was bought on the bargain market Abéché (Tchad) and identified on the basis of 16S RNA (GenBank accession number: OP798035.1). The isolate was cultured on Luria-Bertani (LB) agar for 24 h at 37°C to extract its genomic DNA. After 24 h, a single colony was selected and incubated for 24 h at 37°C in a Luria-Bertani broth until it reached an optical density of 0.6 at 600 nm (OD600) (6). Then, it was submitted to the extraction of genomic DNA using the Wizard Genomic DNA purification Kit (Promega). The genomic DNA concentration was determined by using an Invitrogen Qubit 4.0 fluorometer. Genomic DNA libraries were prepared with the use of the Nextera XT Library (Illumina, San Diego, USA) with 1 ng input DNA kit, accordingly to the manufacturer’s instructions. The sequencing of the entire genome was made by Illumina MiSeq platform using 2 × 250 bp paired-end reads. The raw sequence was processed using Trimmomatic (V.0.39) (7). *De novo* genome assembly was performed using SPAdes (3.11.1 version) (8) and annotated using the National Center for Biotechnology Information-Prokaryotic Genome Annotation Pipeline (NCBI-PGAP) (https://www.ncbi.nlm.nih.gov/genome/annotation_prok/), 6.9 (2025) version (9, 10), via the NCBI genomic submission gate. The search for genes of virulence, resistance to antibiotics, and antimicrobial peptides was performed with VirulenceFinder v2.0 (11), PathogenFinder v1.1 (12), PlasmidFinder v2.1 (13), ResFinder v 4.6.0. (14), and antiSMASH (version 7) (15). Default parameters were used, except where otherwise noted. Biosynthesis of antimicrobial peptide gene clusters, such as bacillilaene, subtilosin A, bacilysin, bacillibactin, surfactin, and fengycin, was identified in the genomic sequence. No coding gene for putative virulence factors, no plasmid, and no antibiotic resistance genes were identified. The genomic sequence was 4,312,934 bp long and spread over 244 contigs. It had a 43.5% GC content and contained a total of 4,703 genes, including 4,588 CDS, 28 rRNAs (11 5S, 10 16S, 7 23S), 82 tRNAs, and 5 ncRNAs.

## Data Availability

The *Bacillus subtilis* ka15 Whole Genome Shotgun (WGS) project has the project accession JBKIWK000000000. The version described in this article is JBKIWK000000000.1. This version of the project (01) has the accession number JBKIWK010000000, and consists of sequences JBKIWK010000001-JBKIWK010000244. The NCBI BioProject accession number is PRJNA1204473. The NCBI BioSample accession number is SAMN46041846.
